# Assessment of Toxic Effects of the Methanol Extract of *Citrus macroptera* Montr. Fruit via Biochemical and Hematological Evaluation in Female Sprague-Dawley Rats

**DOI:** 10.1371/journal.pone.0111101

**Published:** 2014-11-04

**Authors:** Nizam Uddin, Md. Rakib Hasan, Md. Mahadi Hasan, Md. Monir Hossain, Md. Robiul Alam, Mohammad Raquibul Hasan, A. F. M. Mahmudul Islam, Tasmina Rahman, Md. Sohel Rana

**Affiliations:** Department of Pharmacy, Jahangirnagar University, Savar, Dhaka, 1342, Bangladesh; Max-Delbrück Center for Molecular Medicine (MDC), Germany

## Abstract

*Citrus macroptera* Montr. (*C. macroptera*) is locally known as Satkara. The fruit of this plant is used as appetite stimulant and in the treatment of fever. This study therefore aimed to evaluate the toxic effects of the fruit extract using some biochemical and hematological parameters in rat model. The effects of methanol extract of *Citrus macroptera* Montr. fruit administered at 250, 500 and 1000 mg/kg body weight were investigated on hematological and biochemical parameters in Sprague-Dawley female rats. Moreover, histopathological study was performed to observe the presence of pathological lesions in primary body organs. The extract presented no significant effect on body weight, percent water content, relative organ weight and hematological parameters in rat. Significant decrease from control group was observed in the levels of triglyceride, total cholesterol, low density lipoprotein and very low density lipoprotein; thus leading to significant decrease of cardiac risk ratio, castelli's risk index-2, atherogenic coefficient and atherogenic index of plasma at all doses. 500 mg/kg dose significantly decreased alkaline phosphatase (*P*<0.05), 1000 mg/kg dose significantly increased high density lipoprotein cholesterol (*P*<0.05) and 250 mg/kg dose significantly decreased the level of glycated hemoglobin (*P*<0.05) from the control group. There were no significant alterations observed with other serum biochemical parameters. Histopathological study confirmed the absence of inflammatory and necrotic features in the primary body organs. Study results indicate that methanolic fruit extract is unlikely to have significant toxicity. Moreover, these findings justified the cardio-protective, moderate hepato-protective and glucose controlling activities of the fruit extract.

## Introduction

Medicinal plants were and are still one of the major sources of modern medicine. Interest in medicinal plant's pharmacognosy has increased due to using trend of phytotherapy as alternative medicine [1], [2]. Plants produce bioactive compounds that act as defense mechanism against predators and at the same time may be toxic in nature for our health. With the increased interest in the pharmacological activities of the medicinal plants, there is a surge for thorough scientific investigations of these medicinal plants for efficacy and potential toxicity. Proper scientific evidence is necessary to establish the use of plants for medicinal purposes as safe, non-toxic and pharmacologically active.

*Citrus macroptera* Montr., a semi wild species of citrus genus, is known as ‘Satkara’ in Bangladesh. It may be mentioned here that the English meaning of Satkara is ‘Wild orange’. The maximum height of the tree of this fruit is 5 meter. The diameter of the fruit is 6–7 cm. The fruit becomes yellow when it ripens and its rind is thick. As the pulp of this fruit is somewhat dry, it does not have enough juice, which is very sour and a bit bitter [3]. Chowdhury et al. (2008) reported the antioxidant activities of crude extracts of the stem bark of ***Citrus macroptera*** **and isolated** lupeol and stigmasterol [4]. Waikedre et al. (2010) reported the anti-microbial activity of essential oil of ***Citrus macroptera*** **a**gainst five bacteria and five fungi strains. Besides, the author found the presence of beta-pinene as major component [5]. Rana et al. (2012) reported that essential oils obtained by hydro-distillation from the fresh peels of *Citrus macroptera* contained limonene, beta-caryophyllene and geranial as main compounds [6]. Gaillard et al. (1995) isolated edulinine, ribalinine and isoplatydesmine and five aromatic compounds [7]. The people of Bangladesh eat this fruit as a vegetable. The fruit is used as ingredient in cooking different kinds of meat and chicken. Nowadays many Bangladeshi and Indian restaurants offer meat and chicken curries cooked with Satkara. Traditionally, this fruit is used as appetite stimulant and in treatment of fever [8]. In spite of the diverse uses of this fruit, there seems to be a dearth of information about the toxicity of this fruit. Therefore, we have designed our study protocol to evaluate the possible toxicity of the methanolic fruit extract via biochemical and hematological assessment as well as histopathological study in rat model. In addition, the results of the study can provide researchers with the safe levels of the doses of methanolic fruit extract. In this study we used methanol solvent for extraction because this solvent can dissolve various polar bioactive compounds as well as lipophilic compounds. Moreover, it is easy to evaporate this solvent and we get maximum bioactive compounds of the extract by minimizing the loss of compounds due to excessive heat [9].

## Materials and Methods

### Chemicals and reagents

Methanol was bought from SIGMA (Sigma-Aldrich, St Louis, USA). Ketamine inj. was obtained from ACI Pharmaceuticals Ltd., Bangladesh. All clinical diagnostic kits were purchased from Human Laboratories, Germany. All other chemicals and reagents used were of analytical grade.

### Plant material

Fruits of *Citrus macroptera* were collected from Sylhet, Bangladesh and authenticated by an expert Taxonomist. A voucher specimen (Acc. No. 38619) was deposited in Bangladesh National Herbarium for future reference. No field studies were carried out in this study as the fruits were purchased from some local markets of Sylhet, Bangladesh. No specific permissions were required.

### Preparation of plant extract

At first peels were removed. Then the pulp were cut into pieces and then dried in a hot air oven (Size 1, Gallen kamp) at reduced temperature (not more than 50°C) to make suitable for extraction process. Then the dried fruit materials (500 gm) were treated with sufficient amount of pure methanol (1000 ml) for one week at room temperature with occasional shaking (solvent to fruit ratio was 2∶1). The extract was filtered through a cotton plug followed by Whatman No. 1 filter paper. The filtrate was then evaporated under reduced pressure to give a dark green viscous mass and stored at 4°C until it was used. The yield value for the methanol extract was 29.02%.

### Animals and experimental set-up

Sprague-Dawley female rats weighing between 170–230 g were collected from Pharmacology Laboratory, Department of Pharmacy, Jahangirnagar University and were acclimatized to normal laboratory conditions for one week prior to study and were assessed to pellet diet and water *ad libitum*. Temperature of facility was (22±3) °C and light/darkness alternated 12 h apart. The research activities were conducted in accordance with the internationally accepted principles of the US guidelines (NIH publication #85–23, revised in 1985) and following the approval by the Biosafety, Biosecurity and Ethical Committee [Approval Number: BBECJU/N2013(21)] of Faculty of Biological Sciences of Jahangirnagar University, Savar, Dhaka, Bangladesh. The study was carried out in the Department of Pharmacy, Jahangirnagar University.

### Experimental procedure

The animals were assigned to four groups of five animals each. Group-1 was treated as control and group-2, 3 and 4 were treated with 250 mg/kg, 500 mg/kg and 1000 mg/kg doses of methanol extract of *C. macroptera* fruit respectively for 21 days. Different doses of the extract of *C. macroptera* were administered orally by stomach tube. Rats were weighed weekly and observed for behavioral changes, feeding and drinking habits, and general morphological changes. At the end of the 21-day treatment period, after 18 hours fasting, rats from each group were anaesthetized by administration (i.p) of ketamine (500 mg/kg body weight) [10]. Blood samples were collected from post vena cava of rats into EDTA (Ethylene diamine tetra acetic acid) sample tubes for hematological analysis and plain sample tubes for serum generation for biochemical analysis. Serum was obtained after allowing blood to coagulate for 30 minutes and centrifuged at 4000 g for 10 min using bench top centrifuge (MSE Minor, England). The supernatant serum samples were collected using dry Pasteur pipette and stored in the refrigerator for further analysis. All analyses were completed within 24 hours of sample collection [11].

### Hematological analysis

Blood samples were analyzed using established procedures and automated Sysmex KX-21 hematology analyzer. Parameters that were recorded included Hemoglobin (Hb), Red blood cells (RBC), White blood cells (WBC), Platelets, Erythrocyte Sedimentation Rate (ESR), Packed cell Volume (PCV), Mean corpuscular volume (MCV), Mean corpuscular hemoglobin (MCH), Mean corpuscular hemoglobin concentration (MCHC), Pro-calcitonin (PCT), Packed cell volume (PCV), Red cell distribution width - standard deviation (RDW –SD), Red cell distribution width - coefficient of variation (RDW-CV), Platelet larger cell ratio (P-LCR), Platelet distribution width (PDW), Mean platelet volume (MPV), Neutrophils, Basophils, Lymphocytes, Monocytes and Eosinophils.

### Biochemical analysis

Serum samples were analyzed for urea, albumin, total protein, total cholesterol (TC), high density lipoprotein (HDL) cholesterol, triglycerides (TG), uric acid, alkaline phosphatase (ALP), aspartate transaminase (AST) and alanine transaminase (ALT) using Human commercial kits and Humalyzer 3500. Moreover, glycated hemoglobin (HbA1c) test was performed to find out the amount of glycated hemoglobin (HbA1c) using Bio Rad D-10 analyzer. Very low density lipoprotein (VLDL) cholesterol and Low density lipoprotein (LDL) cholesterol concentrations were calculated using the following Friedewald equations [12]: 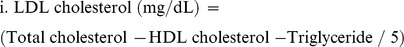


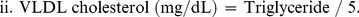



The atherogenic indices were calculated as follows:


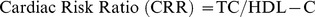
 [13]


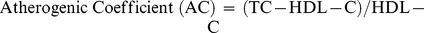
 [14]


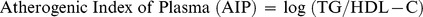
 [15]


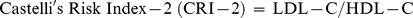
 [16]

### Histopathology

After the collection of blood, all the animals were euthanized for gross pathological examinations of the major organs. The organs such as: hearts, kidneys, lungs, livers, spleens, fallopian tubes, thymuses and ovaries were collected from all the animals for histopathology. The weights of the organs were determined. The relative organ weights were calculated by dividing the individual weight of each organ with the final body weight of each rat. The percent water content was determined by subtracting the dry weight of each organ from the respective weight of each wet organ. Then it was planned to perform histo-pathological examination for the control and high dose group initially. If any histopatholgical findings were observed with high dose group, the mid and low dose groups were to be studied. The selected organs were fixed in 10% neutral buffered formalin, trimmed and a 4–5 µm thickness of tissue sections were stained with hematoxylin and eosin for histopathological investigation using established protocols [17]. The photomicrographs were taken with Olympus DP 72 microscope.

### Data analysis

Data were expressed as mean ± SEM (Standard Error of Mean). Repeated measures ANOVA and One-way ANOVA followed by Dunnett's multiple comparison was performed to analyze data sets. *P*<0.05 was considered significant. Statistical programs used were SPSS (version 16, IBM software Inc, USA) and GRAPHPAD PRISM (version 6.02; GraphPad Software Inc., San Diego, CA, USA).

## Result

### Effect of the extract on the body weight, % water content and relative organ weight of rats

**Figure 1:**
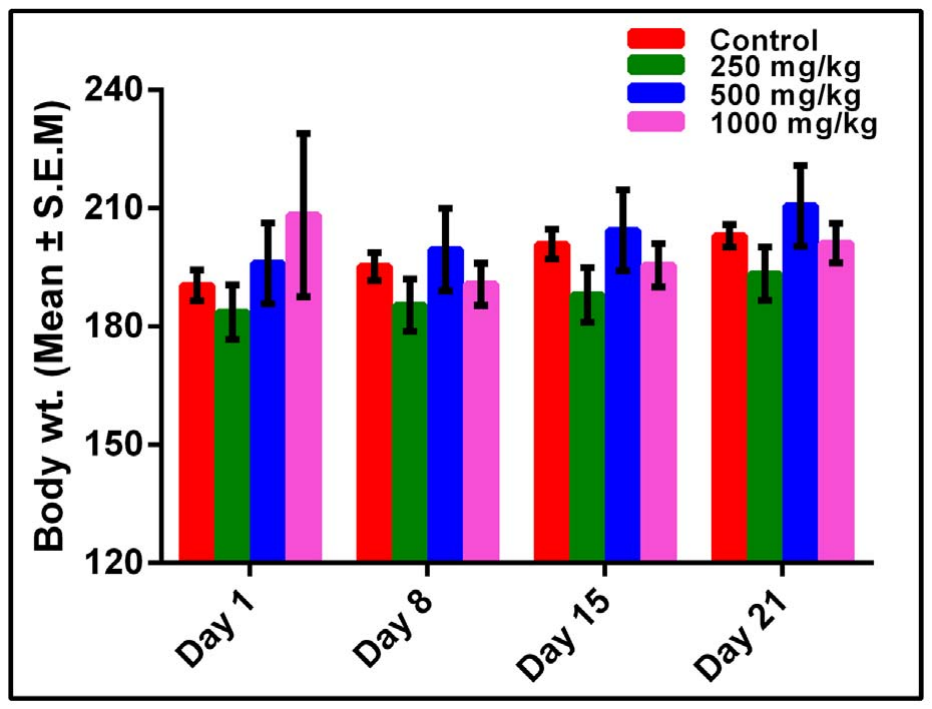
Effect of methanol extract of *C. macroptera* fruit on body weights (g) of Sprague-Dawley female rats in 21 days treatment period. RM ANOVA with Dunnett's multiple comparison was performed to analyze this weight variation in different days. No significant different was found when compared to control group. Time effect was found not significant (*P*>0.05).

There were no signs and symptoms of toxicity and death recorded after three week treatment period. From the figure 1 it is ascertained that body weight did not gradually increase at all doses in comparison with control. There was no significant deviation in weight gain from control at any doses in this three- week treatment period. Table-1 and 2 present effects of different doses of methanol extract on % water content and relative weight of different body organs of rats. No significant difference was noticed in % water content and relative organ weight of primary body organs (Table 1 and 2).

**Table 1:** % Water content of different organs treated with different doses of methanol extract of *C. macroptera* fruit.

Organ	Control	250 mg/kg	500 mg/kg	1000 mg/kg
**Heart**	34.04±1.867	36.121±1.523	34.973±3.011	36.262±2.262
**Kidney**	47.008±1.458	48.565±1.043	48.403±1.011	48.276±2.007
**Lung**	42.271±4.066	51.637±0.481	49.128±2.862	42.75±4.877
**Liver**	58.263±1.185	62.147±1.817	60.882±1.688	61.082±2.068
**Spleen**	38.909±0.820	43.386±2.043	34.971±1.291	34.202±0.887

Values are presented as mean±S.E.M (n = 5). No significant result was found when compared against control. One way ANOVA followed by Dunnett's multiple comparison was performed to analyze this data set.

**Table 2:** Relative Organ weight profile of rats treated with different doses of methanol extract of *C. macroptera*.

Organ	Control	250 mg/kg	500 mg/kg	1000 mg/kg
**Heart**	0.2245±0.002	0.2428±0.007	0.227±0.003	0.243±0.009
**Kidney**	0.2246±0.004	0.2484±0.016	0.231±0.009	0.215±0.020
**Lung**	0.5019±0.067	0.457±0.009	0.4±0.024	0.481±0.041
**Liver**	2.025±0.100	2.387±0.121	2.04±0.08	2.3±0.078
**Spleen**	0.2749±0.008	0.313±0.005	0.314±0.02	0.304±0.013
**Ovary**	0.0235±0.002	0.026±0.001	0.024±0.001	0.025±0.001
**Fallopian tube**	0.2122±0.016	0.2127±0.016	0.204±0.03	0.2±0.026
**Thymus**	0.127±0.007	0.1078±0.011	0.120±0.004	0.110±0.004

Values are presented as mean±S.E.M (n = 5). No significant result was found when compared against control. One way ANOVA followed by Dunnett's multiple comparison was performed to analyze this data set.

### Effect of extract on the hematological parameters

The effects of different doses of the extract on the hematological parameters are tabulated in table 3. No significant difference was found with all hematological parameters.

**Table 3:** Effect of different doses of methanol extract of *C. macroptera* fruit on the hematological parameters of rats.

Hematological parameters	Control	250 mg/kg	500 mg/kg	1000 mg/kg
**ESR(mm)**	5.4±0.748	8±0.836	5.2±1.012	6.6±1.077
**RBC(× 10^12^/L)**	7.016±0.192	6.532±0.245	7.402±0.241	7.388±0.088
**WBC (×10^9^/L**	5.32±0.5	4.95±0.76	4.944±0.421	3.538±0.530
**PLT(× 10^9^/L)**	417.2±43.787	412.8±11.425	468.2±51.361	417±40.707
**Hb(g/dl)**	13.4±0.448	14.125±0.125	13.74±0.430	14.04±0.252
**HCT/PCV(%)**	48.04±1.403	47.56±1.307	47.18±1.383	47.78±1.012
**MCV(fL)**	67.84±1.525	73.62±3.103	63.76±0.78	64.66±0.951
**MCHC (g/dl)**	27.86±0.235	25.72±1.437	29.1±0.23	29.42±0.284
**MCH (pg)**	19.12±0.385	18.86±1.084	18.58±0.162	19.02±0.172
**RDW-SD (fL)**	43.72±1.184	49.275±4.636	38.76±0.62	40.52±1.66
**RDW-CV (%)**	19.66±0.250	21.5±1.662	19.36±0.097	19.46±0.764
**PDW (µm)**	13.56±0.892	14.55±1.12	12.44±0.242	13.04±0.654
**MPV (fL)**	10.3±0.3	11.175±0.45	10.22±0.156	10.24±0.273
**P-LCR**	28.62±2.383	34.275±3.456	27.92±1.151	28.46±1.92
**PCT (µg/L)**	0.432±0.050	0.67±0.106	0.506±0.064	0.428±0.042
**N (%)**	39.48±4.233	39.92±3.222	34.92±0.421	31.725±3.164
**L (%)**	57.84±4.212	53.84±4.84	58.16±2.13	60.06±3.504
**M (%)**	1.66±0.13	4.86±2.345	2.94±0.271	2.94±0.842
**E (%)**	1.02±0.351	1.38±0.871	1.32±0.554	3.675±1.575
**N/L**	0.7218±0.137	0.7892±0.134	0.603±0.017	0.416±0.061

Here N =  Neutrophils, L  =  Lymphocyte, M = Monocyte, E  =  Eosinophils, PLT =  Platelet and N/L = Neutrophil/Lymphocyte ratio. Values are presented as mean±S.E.M (n = 5). No significant result was found when compared against control. One way ANOVA followed by Dunnett's multiple comparison was performed to analyze this data set.

### Effect of extract on the biochemical parameters

Treatment with different doses of methanol extract did not have any significant adverse effect on hepatic biomarker enzymes ALT and AST while 500 mg/kg dose significantly decreased the activity of ALP enzyme. Moreover, three different doses of the extract did not alter the level of total protein and albumin which are major tests to assess liver damage (Table 4). Besides, methanol extract significantly reduced the level of LDL, VLDL, TG, TC, CRR, CRI-2, AC and AIP which are the imperative assays for cardiovascular diseases. Furthermore, 1000 mg/kg dose significantly (*P*<0.05) increased the level of HDL. The determination of uric acid and urea is the biomarker of the normal function of kidney. Insignificant (*P*>0.05) changes were found in the level of these two renal biomarkers. Moreover, 250 mg/kg dose significantly reduced HbA1c (*P*<0.05) whereas in high doses the fruit extract failed to show positive response (Table 4).

**Table 4:** Effect of different doses of methanol extract of *C. macroptera* fruit on biochemical safety parameters in rats.

Biochemical parameters	Control	250 mg/kg	500 mg/kg	1000 mg/kg
**TP(g/dl)**	5.096±0.077	4.544±0.214	5.194±0.156	5.188±0.21
**ALB(g/dl)**	3.82±0.216	3.4±0.124	3.5234±0.090	3.7142±0.186
**GLB(g/dl)**	1.276±0.251	1.1442±0.27	1.671±.150	1.4744±0.144
**A/G**	3.620±0.907	3.9837±1.20	2.1732±0.181	2.6292±0.308
**TG (mg/dl)**	67.222±1.195	46.854±2.928*	36.84±1.501*	34.22±4.94*
**TC (mg/dl)**	75.946±1.964	69.326±0.267*	68.608±0.515*	66.102±0.896*
**HDL (mg/dl)**	56.9498±1.802	59.674±0.6	60.609±0.34	62.17±0.636*
**LDL (mg/dl)**	5.5518±1.097	0.2812±0.134*	0.631±0.301*	0.388±0.061*
**VLDL (mg/dl)**	13.4444±0.241	9.3708±0.585*	7.368±0.3*	6.844±0.987*
**ALT(IU/L)**	49.136±1.634	45.95±0.735	49.48±1.43	46.42±0.852
**AST(IU/L)**	94.54±1.740	90.8±1.870	90.058±0.48	90.248±0.472
**ALP(IU/L)**	361.62±7.891	353.86±15.523	314.63±2.826*	363.5±4.193
**U (mg/dl)**	34.684±2.316	34.716±1.258	38.528±2.647	39.83±2.342
**UA (mg/dl)**	2.0824±0.283	3.0162±0.331	1.7098±0.236	1.4068±0.143
**HbA1c (%)**	2.74±0.16	1.92±0.115*	2.92±0.243	2.72±0.213
**CRR**	1.3353±0.022	1.1622±0.011*	1.132±0.008*	1.0638±0.02*
**CRI-2**	0.097±0.012	0.004±0.002*	0.010±0.005*	0.006±0.001*
**AC**	0.3353±0.022	0.1622±0.011*	0.132±0.007*	0.0638±0.02*
**AIP**	1.042±0.012	0.941±0.017*	0.878±0.009*	0.845±0.033*

TP  =  Total protein, ALB  =  Albumin, GLB =  Globulin, U =  Urea and UA =  Uric Acid. Values are presented as mean±S.E.M (n = 5). One way ANOVA followed by Dunnett's multiple comparison was performed to analyze this data set. * *P*<0.05 when compared against control.

### Histopathological observations

**Figure 2:**
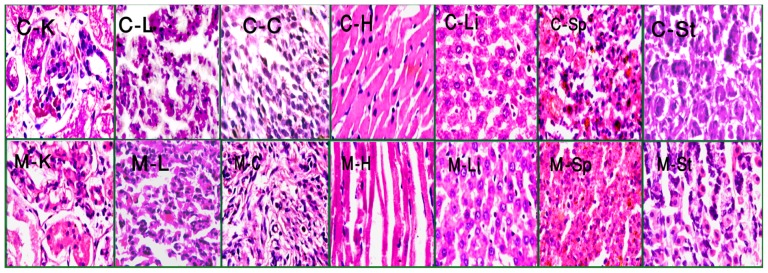
Histopathological photomicrographs of control group and 1000 mg/kg dose group of *C. macroptera* (×100 magnification). No presence of discernible lesions including inflammation and necrosis were observed. Here, C-K =  Control-Kidney, C-L =  Control-Lung, C-C =  Control-Cecum, C-H =  Control-Heart, C-Li =  Control-Liver, C-Sp =  Control-Spleen, C-St =  Control-Stomach, M-K = 1000 mg/kg dose-Kidney, M-L = 1000 mg/kg dose-Lung, M-C = 1000 mg/kg dose-Cecum, M-H = 1000 mg/kg dose-Heart, M-Li = 1000 mg/kg dose-Liver, M-Sp = 1000 mg/kg dose-Spleen and M-St = 1000 mg/kg dose-Stomach.

There were no overt pathological lesions observed both at high and low doses of the methanol extract in histopathological study. In the figure 2 photomicrograph of histopathological study of 1000 mg/kg dose and control were presented. No abnormal signs of toxicity were found in the high dose. Histopathological observations confirmed the normal cellular architecture of the tested organs.

## Discussion

Daily oral doses of the methanol extract up to 1000 mg/kg did not cause any physical abnormalities or death after three week treatment period. In a previous study Uddin et al (2014) performed the acute toxicity of this fruit extract. Administration of doses up to 4000 mg/kg (the highest dose) produced no mortality which was accompanied by normal physical activity of the tested animals [18]. This study strengthens the safety of the extract.

The body of a human being manages a large number of complicated interactions with a view to maintaining balance within a usual range. These interactions make compensatory changes easier which support normal physical and psychological functions. This process is indispensable to the survival of humans and other species. This process is essential to the survival of the person and to our species [19]. 75% weight of the body of an infant and 55% weight of the body of an elderly person are water. It is vital for maintaining cellular homeostasis. Dehydration can cause several physiological disorders [20]. In our study we found no significant alteration in % water content of primary organs from control group which buttresses the claim that the fruit extract does not have toxic effects on normal fluid content and role of body fluid in maintaining homeostasis system. Alteration in the normal weight of the body indicates impairment of normal function of different body organs. Methanol extract did not show any significant alteration on the body weight of the rats compared to control group in 3-week treatment period (Figure 1). It can be suggested that this extract has no negative impact on the body weight of rats treated with three different doses. Relative organ weight may serve as indication of pathological and physiological status in man and animals. Toxic substances induce abnormal metabolic reactions that affect primary organs such as heart, liver, spleen, kidney and lung [21]. Our findings suggest that three doses of the methanol extract of fruit are non-toxic on all vital organs tested in this study. Furthermore, three doses of the fruit extract showed no toxicity on reproductive organs such as fallopian tube and ovary. Therefore, this fruit extract is considered safe for maintaining the normal function of the organs.

Hematological assessment is useful to determine the extent of toxic effects of plant extracts on the blood constituents of an animal [22]. The analysis of blood parameters is closely related to risk evaluation because when tests involve rodents, the hematological system has a higher predictive value of any abnormal toxicity signs and symbols in humans [23]. We found no noticeable hemolytic changes on RBC, Hb, PCV, MCH, MCV, MCHC, RDW-SD and RDW-CV. These findings exclude the possibility of occurrence of anemic condition and other erythrocyte cells related disorders (thalassemia, polycythemia, liver disease, hypothyroidism etc.). Increase in the production of WBC and it's differentials is generally considered to be a marker of stress and a defence mechanism triggered by immune system against various inflammatory conditions (Polymyalgia rheumatica, bacterial infections, hemorrhage, leukemia etc.). The non-significant changes in the level of WBC and differentials including platelet and its indices, neutrophils, lymphocytes, monocytes and eosinophils observed in this study at three doses level suggest the non-toxicity of the fruit extract. Platelet indices are important biomarkers for the early diagnosis of thromboembolic diseases. MPV and PDW are simple platelet indices which are increased during platelet activation [24]. Besides, P-LCR is also considered as an indicator of risk factor associated with thromboembolic ischemic events [25]. A high ESR and PCT indicates that inflammation (Bacterial infections, sepsis, arthritis, etc.) occurs somewhere in the body. Therefore, non-significant impact on the ESR, PCT, WBC and differential counts posits that the fruit extract has no contribution to the induction of infectious diseases and thromboembolic disorders. Moreover, it is safe for hematopoietic system of the body.

Urea and uric acid are the biomarkers of kidney function and retention of these products in the body indicates renal damage [26]–[28]. In this present study, there was no significant increase (*P*>0.05) in the amount of urea and uric acid when compared with the control group. Hence this fruit extract is considered safe and has no destructive effect on normal kidney functions. Elevation in the level of serum transaminase enzymes activities is highly indicative of hepatic impairment in animals [29]. The insignificant change in plasma ALT activity with AST at the doses of 250, 500 and 1000 mg/kg indicates that the extract caused no changes in the liver. ALT is a kind of cytoplasmic enzyme that increases in plasma which is an indication of injuries caused by toxic agents to the liver. Liver injury is characterized by predominant elevation of the ALT and increased activity of mitochondrial enzyme AST in plasma reflects severe tissue injuries [30]. However, 500 mg/kg dose significantly (*P*<0.05) reduced ALP level (table 4) which may account for protective effect on liver disorders. But in 1000 mg/kg the extract did not present significant result. It would indicate selective activity in the median dose and non-selective activity in the highest dose. That is why further research work should be conducted to verify this reason. The level of plasma albumin decreases in response to inflammation [31]. Recent studies have shown that not only albumin concentration but also albumin function are reduced in liver disorders such as liver cirrhosis [32]. The non-significant variation in the level of TP, albumin, globulin and albumin-globulin ratio provides logistic support behind the non-toxic effect of this fruit extract on liver and no link with liver dysfunction.

A high plasma triglyceride level is both an independent and synergistic risk factor for cardiovascular diseases [33] and is often related to hypertension [34], obesity and diabetes mellitus [35]. Elevated total cholesterol level is a familiar and well-known risk factor for developing atherosclerosis and other cardiovascular diseases [36]. High levels of plasma LDL and VLDL cholesterol are responsible for cardiovascular diseases [37] which are accompanied with hypertension and obesity [38]. In this study a very significant decrease was observed in the levels of TC, TG, LDL and VLDL cholesterol which strengthen the lipid lowering activity and represents cardio-protective effect of the fruit extract. Low plasma HDL cholesterol is also a major predictor for cardiovascular diseases [37]. High plasma HDL cholesterol exerts a protective effect by enhancing reverse cholesterol transport through scavenging excess cholesterol from peripheral tissues in our body. 1000 mg/kg dose exhibited very good activity (*P*<0.05) by increasing the level of HDL. This finding further gives strong support for cardio-protective effect of the extract. With these findings (discussed above) in hand we evaluated atherogenic indices which are powerful indicators of the risk of cardiovascular diseases. The higher the values of atherogenic indices the higher the risk of developing cardiovascular disease and vice versa [36]. Methanolic fruit extract reduced three atherogenic indices with significant value *P*<0.05. Moreover. three doses of the fruit extract significantly lowered the value of AIP (*P*<0.05). Hematological parameters MPV and P-LCR are also related to cardiovascular disorders. Higher levels of these parameters coupled with neutrophil/lymphocyte ratio can serve as indication of coronary heart disease [25], [39], [40]. In this study non-significant variation of these parameters from control group provides vital evidences in favor of reduced atherogenic indices and protective effect of the methanolic fruit extract against cardiovascular disorders. Moreover, at low dose fruit extract prevented significant increase in HbA1c in blood. In diabetic condition the level of HbA1c is enhanced due to the production of excessive glucose in blood which further react with blood hemoglobin and creates glycated hemoglobin [41]. Uddin et al (2014) found hypoglycemic activity of the same fruit extract via both *in-vivo* OGTT (Oral Glucose Tolerance Test) model and *in-vitro* α- amylase inhibitory assay, which further substantiated blood glucose level controlling activity [18]. In high doses we did not find any significant HbA1c reduction activity. This may have happened due to the loss of selective activity. That is why further study is required to find out the exact reason. Lipid metabolism abnormality in diabetes is characterized by increase in TC, TG, LDL cholesterol and fall in HDL cholesterol [34]. We recorded significant decrease in the serum levels of these biomarkers which is discussed previously. Moderate activity in decreasing glycated hemoglobin coupled with the reduction of lipid profile proved the efficacy of the fruit extract as moderate hypoglycemic agent and it can control blood glucose level.

Furthermore, up to 1000 mg/kg no discernible lesions including inflammation and necrosis were found. That is why it is logical to infer that methanol extract has no toxic effect on primary body organs when administered at 250, 500 and 1000 mg/kg doses for 3 weeks in rats. Uddin et al, (2014) detected the presence of saponins, steroids and terpenoids in the fruit extract. Therefore, these phytochemicals may exert the non-toxic effect as well as afore-mentioned therapeutic potentials [18].

## Conclusions

In a nutshell, the non-toxic effect evaluated by biochemical and hematological assessment suggests a wide margin of safety for therapeutic doses. The extract has no significant toxic effect on biological parameters. Besides the medicinal uses, we recorded significant cardio-protective effect with moderate hepato-protective and hypoglycemic activities exerted by this fruit extract. These findings justify the therapeutic potential of the fruit. Further research projects should be launched to isolate and characterize active principles and elucidate molecular structures with precise pharmacology.
